# Integration of taxa abundance and occurrence frequency to identify key gut bacteria correlated to clinics in Crohn’s disease

**DOI:** 10.1186/s12866-023-02999-3

**Published:** 2023-09-04

**Authors:** Xunchao Cai, Nan Zhou, Qian Zou, Yao Peng, Long Xu, Lijuan Feng, Xiaowei Liu

**Affiliations:** 1 Department of Gastroenterology and Hepatology, Shenzhen University General Hospital, Shenzhen, 518055 China; 2 Department of Gastroenterology, Xiangya Hospital, Central South University, Changsha, 410008 China

**Keywords:** Crohn’s disease, Mucosal and fecal microbiota, Co-occurrence network, Occurrence frequency

## Abstract

Bacteria abundance alternation in the feces or mucosa of Crohn’s disease (CD) patients has long been applied to identify potential biomarkers for this disease, while the taxa occurrence frequency and their correlations with clinical traits were understudied. A total of 97 samples from the feces and gut mucosa were collected from CD patients and healthy controls (HCs), 16S rRNA-based analyses were performed to determine the changes in taxa abundance and occurrence frequency along CD and to correlate them with clinical traits. The results showed that bacteria communities were divergent between feces and mucosa, while the taxa abundance and occurrence frequency in both partitions showed similar exponential correlations. The decrease of specific fecal bacteria was much more effective in classifying the CD and HCs than that of the mucosal bacteria. Among them, *Christensenellaceae_R-7_group* and *Ruminococcus* were predicted as biomarkers by using random forest algorithm, which were persistently presented (> 71.40% in frequency) in the feces of the HCs with high abundance, whereas transiently presented in the feces (< 5.5% in frequency) and mucosa (< 18.18% in frequency) of CD patients with low abundance. Co-occurrence network analysis then identified them as hub taxa that drive the alternations of other bacteria and were positively correlated to the circuiting monocytes. The loss of specific bacteria in the healthy gut may cause great disturbance of gut microbiota, causing gut bacteria dysbiosis and correlated to immune disorders along CD, which might not only be developed as effective noninvasive biomarkers but also as therapy targets.

**Supplementary Information:**

The online version contains supplementary material available at 10.1186/s12866-023-02999-3.

## Introduction

Crohn’s disease (CD) is one of the inflammatory bowel diseases (IBD) characterized by discontinuous intestinal injury and inflammation, which may spread across the entire gastrointestinal (GI) tract [1]. In severe situations, transmural lesions, including granulomata, deep fissuring ulcers, and lymphoid distribution accumulate [2, 3]. It has been reported that the highest incidence of CD in North America, Europe, and Asia/Middle East was 20.2, 12.7, and 5.0 per 100,000 people / year, respectively [4, 5]. With its high incidence in developed countries and fast increasing in developing countries, it is currently a worldwide health issue [6].

Although great efforts have been made to determine the pathogenesis, no solid or determined conclusions have been reached to fully interpret the etiology. Currently, it is widely accepted that the pathogenesis of CD is multifactorial and involves the interplay of genetics, immune dysregulation, and environmental factors [7, 8]. Among these factors, the disturbance to the gut microbiota, i.e., dysbiosis, especially bacteria, has been recognized, playing a critical role in convergent studies, which hypothesized that abnormal immune response to gut microbiota dysbiosis resulted in recurrent intestinal inflammation in genetically predisposed individuals [9, 10]. Patients with CD commonly have altered gut microbiota assemblages compared to healthy controls (HCs), characterized by decreased bacterial diversity and alternations in specific bacteria abundance [11, 12]. For example, the biodiversity and the relative abundance of Firmicutes are decreased while those of Proteobacteria are increased in CD patients [13]. Commonly, the decreased taxa are mainly short-chain fatty acid producers such as *Faecalibacterium prausnitzii*, *Lactobacillus*, Erysipelotrichaceae, and Bifidobacteriaceae [14, 15], while the increased taxa are mainly proinflammatory bacteria such as *Fusobacterium* and *Escherichia* members [16]. However, most of these studies determined the luminal/fecal microbiota dysbiosis by abundance and diversity alternations in the feces, which underestimated the frequency and occurrence of specific taxa in the disease. Moreover, comparable dysbiosis at the mucosal surface, either in un-inflamed mucosal areas or at sites of inflammation, has rarely been investigated [1]. A few recent studies have shown that the microbiota assemblages in the rectum, ileal, or colon mucosa display a unique microbial signature compared to that in fecal samples [17–20]. Some mucosa bacteria were found to be predictive for CD recurrence or newly diagnosed and treatment-naïve CD. In recurrent CD, researchers discovered that the abundance of γ-proteobacteria, Corynebacterium, and *Ruminococcus gnavus* increased, while *Ruminoclostidium 6* decreased [21]. It should be noted that *Ruminiclostridium* was found to be depleted in the mucosa of newly diagnosed and treatment-naïve CD patients [22]. Thus, both the luminal/fecal and mucosal microbiota have the potential for the prediction, diagnosis, or treatment of CD. However, it is unclear which parts are most closely related to CD, or whether they could work together to drive the occurrence of CD and the response to abnormal immune regulation.

Due to the multifactorial pathogenesis of CD, clinical tests and Crohn’s Disease Activity Index (CDAI) evaluation were commonly performed for the primary diagnosis of CD, combining with imaging by endoscopy and histological traits [23]. While the correlations between the clinic and microbiota alternation were not well indicated. In order to systemically link changes in the fecal microbiota and mucosal microbiota with the clinical traits of CD, here in this study, a total of 97 samples from the feces and gut mucosa of CD patients and HCs were collected and 16S rRNA amplicon sequencing was performed to determine the microbiota assemblage patterns. The microbiota assemblage patterns of the feces and mucosa were explored, and machine learning using a random forest algorithm was adopted to construct the prediction model for CD. The microbiota was then clustered as co-occurrence modules/clusters using the weighted correlation network analysis (WGCNA) to correlate with clinical traits, identifying potential non-invasive biomarkers for CD and deciphering possible mechanisms for the etiology of CD.

## Materials and methods

### Study Population

All CD patients and HCs in this study were enrolled by the Department of Gastroenterology, Xiangya Hospital, Central South University, from July 2018 to May 2019. The study was approved by the Ethics Committee of Xiangya Hospital, Central South University, and written informed consent was obtained from all participants prior to enrollment. Besides, sample collection from the participants was approved by the Research Ethics Board of the Xiangya Hospital of Central South University. All patients met the diagnostic criteria for CD and were followed up for at least 6 months, the disease phenotype and activity were determined according to the Montreal classification system [24]. Exclusion criteria included (1) those who were unable to provide informed consent, (2) presence of comorbidities of the biliary tract or liver disease, (3) administration of antibiotics or cathartics four weeks before sample collection [25], (4) allergy to fluorescein, pregnancy or breastfeeding, and (5) acute gastrointestinal bleeding. The clinical tests, including complete blood counting, erythrocyte sedimentation rate, and C-reactive protein (CRP) levels were performed by the Department of Clinical Laboratory in Xiangya Hospital using standard methods. CDAI was evaluated for each CD participant as well to assess the disease activity. The degree of anxiety and depression of all participants were evaluated using the Self-rated Anxiety Scale (SAS) [26] system and the Self-Rated Depression Scale (SDS) [27] system, respectively. HCs were enrolled with age and gender matched to the CD group, and passed the exclusion criteria.

### Sample collection and sequencing

Approximately 0.5 g of fresh fecal samples from CD patients and HCs were collected in a 2.0 mL sterile falcon tube, which was immediately transferred to liquid nitrogen and stored at -80 ° C until further processing. Colonoscopy was performed using an Olympus Exera II GIF HsheshiduQ190 or enteroscope SIF-Q180 (Olympus Europa GmbH, Hamburg, Germany). The mucosa sample of each CD patient was collected in sterile cryovials, including biopsies of the inflamed area (Inf_M) and the uninflamed area (Uinf_M) (approximately 5 cm from the inflamed area), two inflamed and two uninflamed biopsies were taken per individual. Meanwhile, one from each was sent for histological analysis, the others were transferred directly into liquid nitrogen and stored until DNA extraction. The total genomic DNA of the fecal samples was extracted using the TIANgen Stool DNA Kit (TIANGEN, Beijing, China), according to the manufacturers’ instructions. Total genomic DNA from mucosal biopsies was extracted using the FastDNA® SPIN Kit for Soil (MP Biomedicals, LLC, Illkirch, USA), according to the manufacturer’s instructions. DNA quality and purity was robustly determined using a NanoDrop ND-1000 Spectrophotometer (Thermo, Massachusetts, USA). The quality-controlled DNA was outsourced to Novogene Company (Nanjing, China) to construct 16 S rRNA sequencing libraries (V3 and V4) and then sequenced using the PE250 strategy on the Illumina platform. QIIME2 (version 2021.2) was used for raw reads filtering and quality control. The DADA2 implanted in QIIME2 was used to denoise the data and produce amplicon sequence variants (ASV), and the taxonomic annotation was performed based on the ‘silva-138-99-nb classifier’ pre-trained in QIIME2. Further data visualization was performed using R (4.0.2).

### Statistics of bacterial assemblages

The Spearman’s ranked correlation method and a significance test of 999 permutations were used to determine the correlations between the alpha diversity indices and clinical traits, a *p-value* ≤ 0.05 and the absolute value of the correlation coefficient ≤ 0.5 were designated as significantly correlated pairs. The two-sided Welch test was used to determine differences in taxonomic abundance between different groups. Unless otherwise stated, a *p*-value ≤ 0.05 was considered significant. Generally speaking, the core taxa in half-closed artificial systems are persistent and high in abundance, while the satellite species are transient and low in abundance [28]. To better understand the patterns of bacterial assemblages in the gut (i.e., the fecal/luminal or mucosal microbiota), we define the bacterial community into the following three ecological categories-based taxa occurrence frequency: persistent (≥ 75% of samples), intermittent (25 ~ 75% exclusive), and transient (≤ 25% of samples) [29].

### Prediction model construction and network analysis

A machine learning model was constructed for data group prediction and key taxa mining using the randomforest R software package randomforest. The “mtry” value was determined when the average error rate was the lowest, and the “ntree” was determined when the model was stable at the smallest tree counts. Moreover, WGCNA was conducted to find taxa clusters/modules highly correlated by using the R software package “WGCNA”, to correlate the bacterial clusters to one another and to clinical traits. The WGCNA was conducted according to the software manual [30]. In detail, the abundance matrix of taxa containing all samples was first clustered using a hierarchy clustering function implanted in WGCNA to check if there were outliers, which would be removed in further analysis. Finally, the dynamic tree-cut method was used to identify the co-occurrence taxa modules of the whole microbiota, in which the soft-power was determined to be 10, and the minimum taxa module size was set to 30. Then, Cytoscape v3.7.1 was used for network visualization and topological analysis [31, 32].

## Results

### Divergent microbiota structure between different pathology or physiology groups

**Fig. 1:**
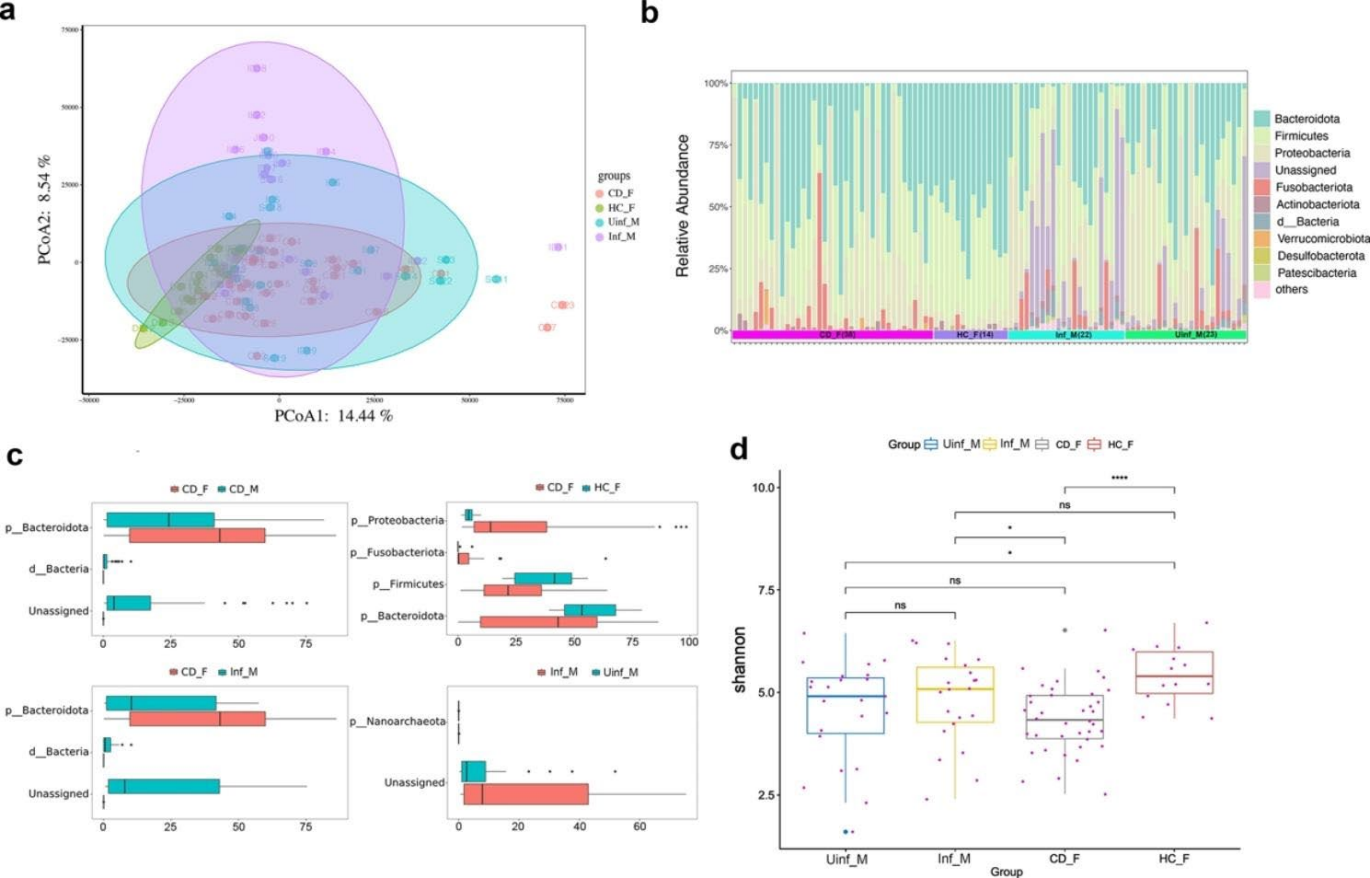
Differences between pathology or physiology groups in specific taxa and alpha diversity. **(a)**. β-diversity between the mucosa and feces in HCs and CD groups indicated by constrained analysis of the principal coordinates on the Bray-Curtis distance. **(b)**. Bar plots representing the relative taxa abundance of each sample at the phylum level. **(c)**. Differences between pathology or physiology groups were calculated based on the relative abundance of taxa at the phylum level (Two-sided Welch’s test, *p*-value ≤ 0.05 was considered significantly different, and only taxa showing differences greater than 0.1% were plotted in the figure). **(d)**. Differences between pathology groups displayed by the Shannon alpha-diversity index. ^*^, *p*-value ≤ 0.05; ^**^, *p*-value ≤ 0.01; ^***^, *p*-value ≤ 0.005; ^****^, *p*-value ≤ 0.001; ns, *p*-value > 0.05, not significantly different. We defined pathology groups as samples from same category of body components but with different pathologic attributes (i.e., feces samples from CD patients (CD_F) or HCs participants (HC_F) and gut mucosal samples from inflamed area or uninflamed area, including HC_F vs. CD_F and Inf_M vs. Uinf_M); physiology groups from gut mucosa but from different region of anatomy (i.e., samples from the feces or gut mucosal samples (CD_M), including CD_F vs. CD_M, CD_F vs. Inf_M and CD_F vs. Uinf_M).

Demographic and clinical characteristics were listed in Table S1. Firstly, we confirmed that the sequencing depth is enough to represent the bacterial assemblages from the fecal and mucosal samples (Fig. S1). Constrained analysis of principal coordinates using Bray-Curtis distance displayed that bacteria communities from the HCs feces were clustered separately from those of the CD feces and mucosa, and the fecal bacteria communities of CD patients were divergent from those of the mucosa (Fig. 1a). These differences were confirmed to be significant using pairwise PERMANOVA analysis (*p*-value ≤ 0.05) (Fig. S2). For the mucosal bacteria communities, high heterogeneity was observed within the group (i.e., Inf_M or Uinf_M) (Fig. 1a and b).

The top ten abundant taxa were Bacteroidota, Firmicutes, Proteobacteria, etc. (Fig. 1b). Among them, Bacteroidota, Firmicutes, and Proteobacteria were absolutely the most dominant, with a cumulative abundance higher than 85%. A significantly higher abundance of Unassigned taxa and d_Bacteria was observed, while a lower abundance of Bacteroidota was observed in the mucosa (e.g., CD_M and Inf_M) than that in the feces (i.e., CD_F) in CD patients (Fig. 1c). As for the feces, the abundances of Proteobacteria and Fusobacteriota were increased, while those of Firmicutes and Bacteroidota were decreased in CD (Fig. 1c). Furthermore, the HCs showed the highest while the CDs showed the lowest alpha diversity when compared to other groups (Kruskal-Wallis test, *p*-value ≤ 0.05) (Fig. 1d). No significant differences in alpha diversity were observed between the inflamed mucosa and uninflamed mucosa (Fig. 1d), while they were actually shaped by divergent taxa with high heterogeneity as displayed in Fig. 1a and b.

### Gut microbiota assemblages modeled by occurrence and abundance

**Fig. 2:**
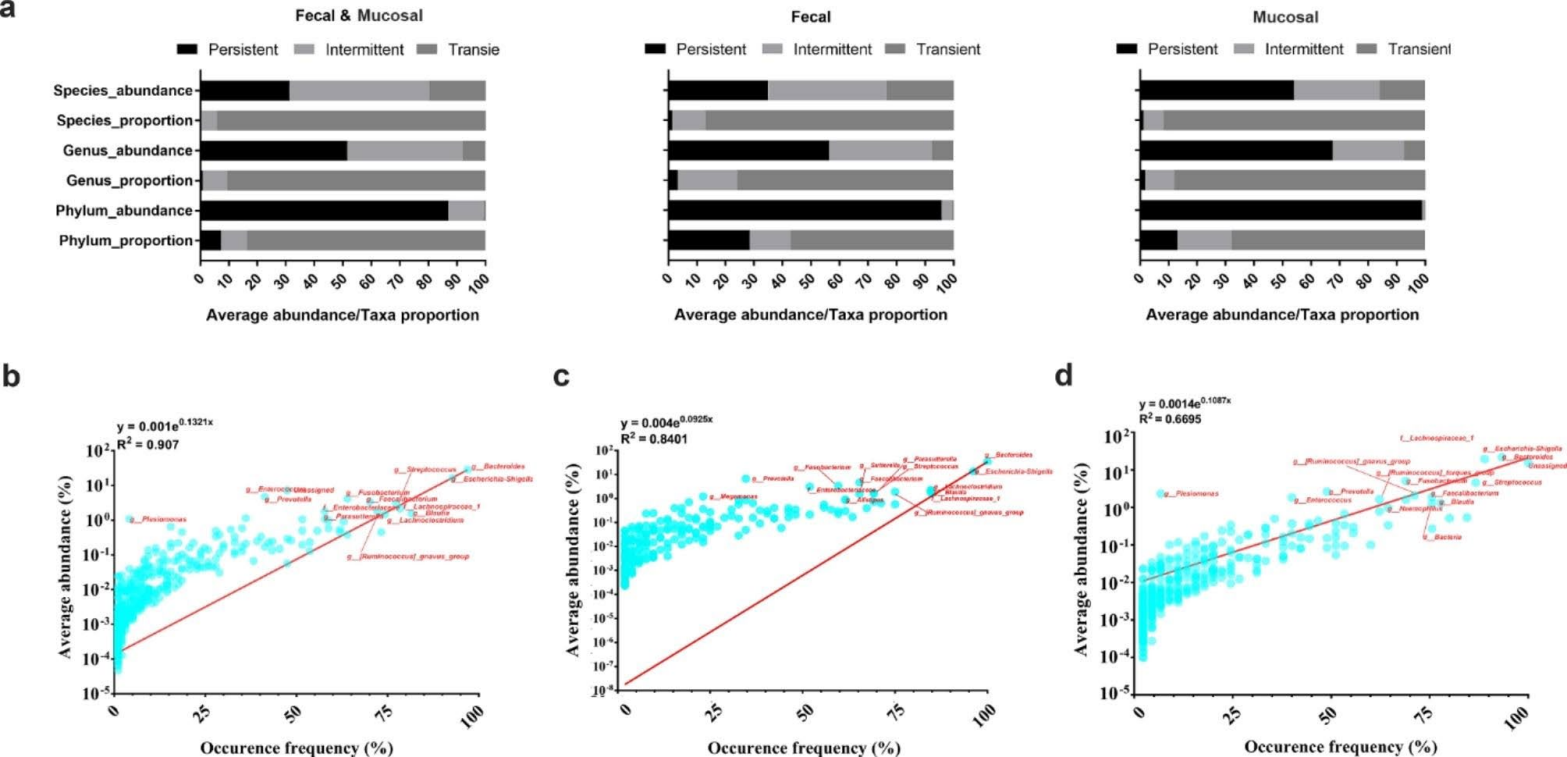
Characterization of bacteria assemblage patterns based on taxa abundance and occurrence frequency model. **(a)**. The taxa ecological category, taxa proportion to all detected taxa, and the average abundance of the taxa ecological category at different taxonomic levels (i.e., phylum, genus and species). **(b), (c)**, **(d)**, The best fitted model for average taxa abundance and occurrence frequency of different ecological communities (b: Fecal and Mucosal community; c: Fecal community; d: Gut Mucosal community)

We divided the samples into three ecological groups (group-FM: Fecal and Mucosal combined community; group-F: Fecal community; group-M: Mucosal community) to globally view the gut microbiota assemblages. By classifying each group into an ecological category, we found that four phyla (i.e., Bacteroidota, Firmicutes, Proteobacteria, Actinobacteriota) were classified as persistent taxa in group-FM (Fig. 2a). Although they only took a proportion of 7.27% (4/55) of all detected phyla in this community, the total abundance of these four phyla was 87.05% (Fig. 2a). The intermittent phyla in group-FM were Unassigned, Fusobacteriota, d_Bacteria, Desulfobacterota, and Patescibacteria (total abundance: 12.37%; proportion: 9.09% (5/55)). From a global point of view, persistent and intermittent taxa (9/55) in group-FM took absolute dominant positions in this community due to their high cumulative abundance (99.41%). This assemblage pattern could also be observed in the fecal (group-F) or mucosal (group-M) community, where the total abundance of the persistent and intermittent taxa took a low proportion of the detected taxa but with a high cumulative abundance (> 75%) (Fig. 2a). Besides, *Bacteroides*, *Escherichia-Shigella* and *Blautia* were displayed as shared persistent genera between the three defined communities (Fig. 2b and c, and 2d). The positive correlations between the taxa abundance and occurrence frequency in these three defined communities were further found to be best fitted by the exponential formulas (Fig. 2b and c, and 2d).

### Machine learning based methods to identify key taxa

**Fig. 3:**
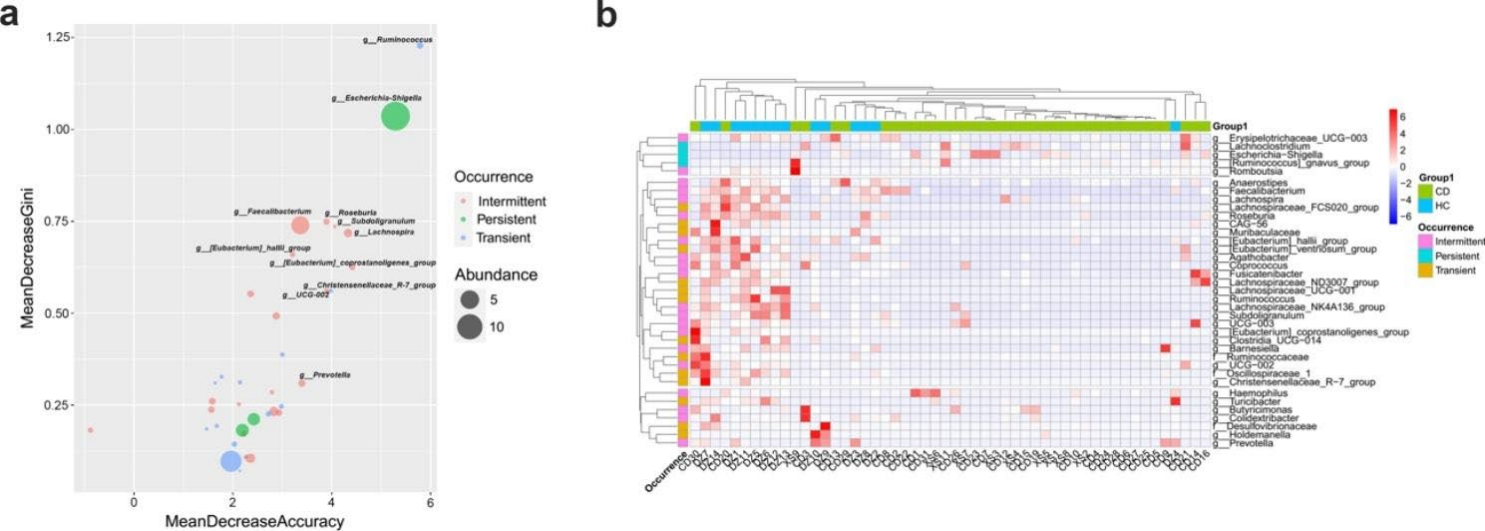
Potential important genera biomarkers in the feces to classify CD or HCs. A random forest algorithm was used to construct a machine learning model to classify CD patients and HCs. **(a)**. MeanDecreaseAccuracy and MeanDecreaseGini of the top 30 genera in the CD/HCs classification model. **(b)**. Cluster and heatmap display of the top 30 key genera in the CD/HCs classification model

As taxa of the three ecological categories at genus level showed very similar cumulative abundance in each community (Fig. 2a), we chose the genus abundance data to construct the machine learning model based on the random forest algorithm. For the mucosal communities (inflamed mucosa vs. uninflamed mucosa), the lowest out-of-bag (OOB) error rate was 44.44%, indicating that there were no effective genera to classify inflamed mucosa and uninflamed mucosa (Fig. S3a). Of note, the model to classify fecal microbiota between CDs and HCs showed excellent performance with an OOB error rate of 11.54% and 100% accuracy to predict the practical data (Table S2, Fig. S3b). The key genera in the CD/HC classification model are shown in Fig. 3a. Among the top ten genera, except *Escherichia- Shigella* increased in CD feces, all others decreased (Fig. 3b). In addition, only one of them was a persistent genus, two were transient genera, while seven were intermittent genera (Fig. 3b). These key taxa belong to three phyla and three classes, and most of them are Clostridia (Table 1). Notably, *Ruminococcus*, *Christensenellaceae_R-7_group*, *[Eubacterium]_coprostanoligenes_group* and *UCG-002* represented in low frequency in the CD feces but high occurrence in that of HCs, which could be developed as effective diagnostic biomarkers (Table 1).

**Table 1:** Characteristics of the top ten key fecal taxa to separate CD patients and HCs

Phylum	Class	Order	Family	Genus	Occurrence frequency			
					CD_F	HC_F	Uinf_M	Inf_M
Proteobacteria	γ-proteobacteria	Enterobacterales	Enterobacteriaceae	*Escherichia-Shigella*	100% (38/38)	85.71% (12/14)	91.30% (21/23)	86.36% (19/22)
Bacteroidota	Bacteroidia	Bacteroidales	Prevotellaceae	*Prevotella*	23.68% (9/38)	64.29% (9/14)	52.17% (12/23)	45.45% (10/22)
Firmicutes	Clostridia	Oscillospirales	Ruminococcaceae	*Faecalibacterium*	52.63% (20/38)	100% (14/14)	86.96% (20/23)	63.64% (14/22)
Firmicutes	Clostridia	Lachnospirales	Lachnospiraceae	*Roseburia*	15.79% (6/38)	85.71% (12/14)	17.39% (4/23)	40.91% (9/22)
Firmicutes	Clostridia	Oscillospirales	Ruminococcaceae	*Subdoligranulum*	23.68% (9/38)	85.7% (12/14)	43.48% (10/23)	36.36% (8/22)
Firmicutes	Clostridia	Oscillospirales	Ruminococcaceae	*Ruminococcus*	5.26% (2/38)	78.57% (11/14)	13.04% (3/23)	18.18% (4/22)
Firmicutes	Clostridia	Oscillospirales	[Eubacterium]_ coprostanoligenes_group	*[Eubacterium]_* *coprostanoligenes_group*	10.53% (4/38)	85.71% (12/14)	13.04 (3/23)	18.18% (4/22)
Firmicutes	Clostridia	Oscillospirales	Oscillospiraceae	*UCG-002*	10.53% (4/38)	78.57% (11/14)	34.78% (8/23)	40.91% (9/22)
Firmicutes	Clostridia	Lachnospirales	Lachnospiraceae	*Lachnospira*	18.42% (7/38)	85.71% (12/14)	17.39% (4/23)	22.73% (5/22)
Firmicutes	Clostridia	Christensenellales	Christensenellaceae	*Christensenellaceae_R-7_group*	5.26% (2/38)	71.43% (10/14)	13.04 (3/23)	18.18% (4/22)

### **Characterizing the co-occurrence of clinical-related taxa modules**

**Fig. 4:**
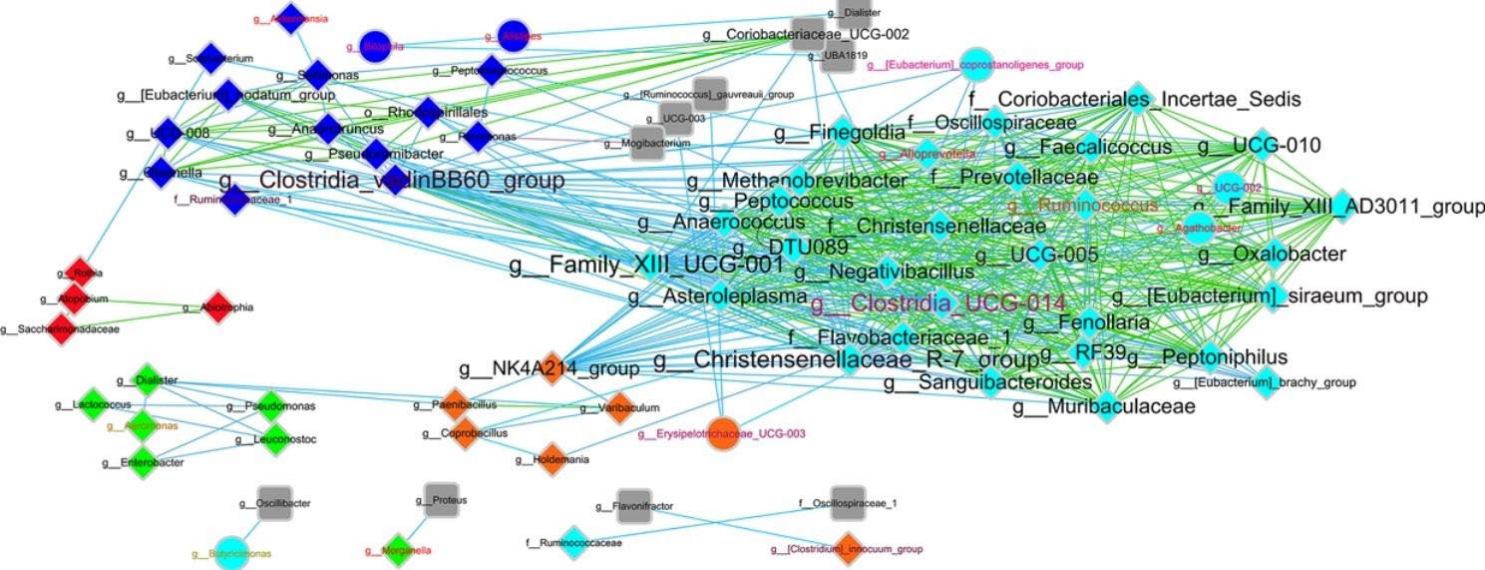
Visualization of the networks of the clinically relevant cooccurrence taxa modules. Different source node colors (i.e., red, blue, green, brown, and turquoise) in the networks represent different genus modules. The node label size in the network represents the connectivity of it, the bigger the node label size, the more the connection is. Node label color and edge color represent the average abundance of each genus and correlation coefficient between two nodes, the darker the color is, the lower the abundance and correlation, respectively. Target nodes were marked with a grey color but not the corresponding module colors

To further clarify the correlations between specific taxa groups and clinical traits, WGCNA was conducted using the genus abundance of CD feces. After removing missing values and outliers (Fig. S4), a total of 189 genera in the fecal bacteria community were finally separated into eight taxa modules, colored gray, blue, brown, green, yellow, red, black, and turquoise (Fig. S5). Among these taxa, five modules were significantly correlated with clinical traits, i.e., module green and module brown negatively correlated with serum glucose and complement C4 (CC4), while module red, module brown, module blue, and module turquoise positively correlated with CRP, basocyte ratio (bas_ratio), serum complement C3 (CC3), and circulating monocytes, respectively (Fig. S5). Of the machine learning identified key genera to classify CD patients and HCs, six were significantly correlated to clinics, of which one (*Escherichia-Shigella*) clustered into module red, positively correlated to CRP; five (*Christensenellaceae_R-7_group*, *Prevotella*, [*Eubacterium*]_*coprostanoligenes* _group, *Ruminococcus* and UCG-002) clustered into module turquoise, positively correlated to circulating monocytes, and decreased in CD. The co-occurrence network further displayed that *Christensenellaceae_R-7_group*, [Eubacterium]_*coprostan*, *oligenes*_group, and *Ruminococcus* showed positive correlations with other genera in module turquoise, especially for *Christensenellaceae_R-7_group, which* showed high connectivity and being a hub taxon in the network, might play vital roles in the formation of this network. Besides, f_Christensenellaceae also showed high connectivity (Fig. 4). However, these hub taxa identified from the feces did not show significant differences between the inflamed mucosa and uninflamed mucosa (Fig. S6), which suggests that they might not be the direct cause of the inflammation.

## Discussion

In this study, we found that divergent microbiota groups formed between different pathology (e.g., CD feces vs. HC feces) or physiology groups (e.g., CD feces vs. CD mucosa, inflamed mucosa vs. uninflamed mucosa). Although no significant differences in alpha diversity or beta diversity were observed between the inflamed mucosa and the uninflamed mucosa, a much higher abundance of Unassigned/d_Bacteria taxa was found in the inflamed mucosa. Taxa abundance distribution (TAD) patterns in both the gut lumen and mucosa revealed that taxa average abundance and occurrence frequency were fitted by exponential correlations. Machine learning based methods combined with taxa occurrence analysis revealed that the loss of specific taxa of Clostridia in the HC feces was excellent to classify HC and CD. Co-occurrence taxa modules further disclosed that *Ruminococcus* and *Christensenellaceae_R-7_group* represented in low occurrence in the CD feces but high occurrence in that of HCs might drive the alternation of bacteria taxa in CD feces and resulted in the disturbance of gut immune hemostasis. This study revealed that more attention should be paid to the occurrence of specific taxa in the HC feces, which might be helpful to develop novel diagnostic markers and to find the pathogenesis behind the microbiota dysbiosis in CD.

Previous studies have well documented that patients with CD display fecal microbiota dysbiosis compared with healthy controls, particularly with respect to reduced microbial diversity and alternated taxa abundance [10]. The most common findings are the decreased abundance of Firmicutes (e.g., *Faecailbacterium prausnitzii*), and the increased abundance of Proteobacteria (e.g., *Escherichia coli*) [33, 34]. Consistent with previous studies, similar taxa abundance alternations and reduced alpha diversity were observed in CD feces in this study (Fig. 1b). Meanwhile, divergent microbiota assemblages between physiology groups (e.g., feces versus mucosa) were observed (Fig. 1). Furthermore, several studies using colonic mucosa from CD patients reported a neutral diversity result when compared to controls [35]. In a recent study, Olaisen et al. (2021) reported that the microbiota assemblage is similar in the inflamed and proximal uninflamed ileal mucosa, and that neither ileal sublocation nor endoscopic inflammation influences the mucosa-associated microbiota [1]. We found that the mucosal microbiota of the inflamed or proximal un-inflamed did not show significant differences (Fig. 1d). However, this does not mean there are no differences between them when referred to specific taxa, Unassigned-taxa and d_Bacteria were observed to be highly represented in the inflamed mucosa of the CD patients (Fig. 2), their association with CD should be considered seriously and more efforts should be made to clarify their taxon assignments and functions.

To date, associations between the microbiota and CD focus on describing the alternations of abundance or diversity, which underappreciates the occurrence frequency of specific taxa in each community. Zhang et al. (2012) reported that the top 25.5% of the detected genera represented 89.1% of the abundance in the microbial communities of activated sludge from 14 wastewater treatment plants [35]. This pattern of assemblage of microbiota has also been observed in many other studies [36, 37], although not discussed in the GI tract system. The TAD analysis showed that 16.36%, 9.47%, and 5.94% of the detected taxa had cumulative abundances of 99.41%, 92.00%, and 80.26% at the phylum, genus, and species level, respectively, in the GI ecosystem (Fig. 2a), indicating that the gut microbiota was assembled similarly to other ecosystems. Moreover, the best model of taxa abundance and frequency of occurrence in the feces and mucosa was determined to fit for exponential correlations (Fig. 2b and c, and 2d), which have been found in other communities [29, 35]. We found that seven of the top ten key taxa identified by the machine learning method were intermittent taxa, implying that taxa presence/absence but not abundance could be more relevant to CD. The low presence of specific taxa such as *Ruminococcus*, *Christensenellaceae_R-7_group*, *[Eubacterium]_coprostanoligenes_ group*, and *UCG-002* in the feces of CD patients but the high presence in that of HCs made them a high potential to be developed as useful diagnostic biomarkers in the future. Nevertheless, these key taxa showed no significant changes between the inflamed and uninflamed mucosa (Fig. S6), implying that their correlation with CD may not be due to their colonization on the mucosa.

Furthermore, we identified five taxa modules that were significantly correlated with clinical traits (Fig. S5). In particular, modules turquoise and red, containing five and one of the machine learning identified taxa, were positively correlated to monocytes and CRP, respectively (Fig. 4). The monocytes could respond to signals from the local microenvironment, maintaining immune homeostasis by their hypo-responsiveness to bacterial stimulation and promoting local regulatory T-cell proliferation. However, under acute intestinal inflammation, this homeostasis is disturbed, which can induce a cascade of inflammatory immune responses and result in chronic inflammation [38–41]. Solid proof of the correlation between the turquoise module taxa and monocytes has not been reported, but their correlation to CD has been widely appreciated. For example, the monocyte compartment has been found to play dual functions in CD, the inadequacy of which on one hand, initiates the disease, whereas its overactivity also maintains the colitis [42]. Ruminococcaceae (e.g., [*Eubacterium*]_*coprostanoligenes*_ group, *Ruminococcus*) and *Prevotella* are short-chain fatty acid (SCFA) producers that are commonly decreased in CD patients [21, 43]. *UCG-002* belongs to the Oscillospiraceae family, which is a well-known producer of valeric acid and is positively correlated with anti-inflammatory [44]. Christensenellaceae have been observed abundantly in the feces of healthy people but absently in those of CD patients [21]. On the contrary, the taxa in module red could be pro-inflammatory bacteria, as they are positively correlated with CRP. *Escherichia-Shigella* belonging to module red has been identified as proinflammatory bacteria (e.g., adherent invasive *Escherichia coli* (AIEC)) that induce the Th17 response, improve TH1 cell accumulation and promote proinflammatory cytokines and fibrotic growth factors [45, 46]. Consequently, the increasing level of *Escherichia-Shigella* clusters may trigger acute inflammation in the gut mucosa, resulting in the disturbance of immune homeostasis and inducing chronic inflammation, while decreased taxa such as *Ruminococcus* and *Christensenellaceae_R-7_group* could be the cause of the increased *Escherichia-Shigella* levels as they are presented as hub taxa in the co-occurrence networks (Fig. 4). Although other key taxa such as *Lachnospira* and *Faecalibacterium* were not identified correlating to the clinical traits tested, strains belonging to these two genera are well known for their anti-inflammatory properties by producing SCFA, which suppresses inflammation and alleviates colitis by regulating macrophage M2 and regulatory T cells [47].

In conclusion, the bacteria assemblages were divergent between feces and mucosa in CD patients. Although similar diversities were observed between the inflamed mucosa and the uninflamed mucosa, the highly represented unassigned taxa in the inflamed mucosa should not be neglected. In addition to the abundance of taxa, more attention should be paid to the occurrence of specific taxa in the gut microbiota of CDs and HCs, especially those persistent in HCs but transient or absent in CDs, and significantly correlated to clinical traits. More importantly, the integration of the gut microbiota and clinical traits would be helpful in interpreting the real roles of specific taxa in CD. Due to the inaccessibility of biopsies, no mucosa was collected from HCs in this study. These limitations will be fixed in our further long-term studies. Although there are limitations, this study provides novel insights into studying specific taxa in CD, paying attention to the frequency of occurrence of the taxa and their correlation with clinical traits.

### Electronic supplementary material

Below is the link to the electronic supplementary material.

Supplementary Material 1

## Data Availability

The raw reads datasets generated for this study can be found in the Short Read Archive (SRA) database: https://www.ncbi.nlm.nih.gov/sra/PRJNA800628.

## Abbreviations

SAS: Self-Rated Anxiety Scale
SDS: Self-Rated Depression Scale
BMI: Body Mass Index
CDAI: Crohn’s Disease Activity Index
HCT: hematocrit
neu_ratio: neutrocyte ratio
lym_ratio: lymphocyte ratio
bas_ratio: basophil ratio
eos_ratio: eosinophil ratio
mon_ratio: monocyte ratio
ery_size: erythrocyte size
MCH: Mean Corpsular Hemoglobin
MCHC: Mean Corpsular Hemoglobin Concentration
RDW: Red-cell Distribution Width
PCT: Platelet Cell Thrombocrit
MPV: Mean Platelet Volume
TP: Total Protein
A/G: Albumin/Globulin
TBIL: Total Bilirubin
DBILI: Direct Bilirubin
TBA: Total Bile Acids
ALT: Alanine Transaminase
AST: Aspartate Aminotransferase
PT: Prothrombin Time
P-Thr-Per: Prothrombin Percentage
INR: International Normalized Ratio
APTT: Activated Partial Thromboplastin Time
PTV: Prothrombin Time
FG: Fibrinogen
FDPs: Fibrin Degradation Products
PLG-Ag: Plasminogen Antigen
PLG-III-Ag: Plasminogen III Antigen
CRP: C Reactive Protein
ESR: Erythrocyte Sedimentation Rate
CC4: Complement C4
CC3: Complement C3
IgG: Immunoglobulin G
IgA: Immunoglobulin A
IgM: Immunoglobulin M
