# Supplementary material for: Integration of taxa abundance and occurrence frequency to identify key gut bacteria correlated to clinics in Crohn’s disease
Source: BMC Microbiol. 2023 Sep 4;23:247. doi: 10.1186/s12866-023-02999-3 (PMC10476393; doi:10.1186/s12866-023-02999-3)
Supplement: Supplementary file 1 — Supplementary Material 1 [file 12866_2023_2999_MOESM1_ESM.docx]

**Integration of taxa abundance and occurrence to identify** **gut bacteria correlated to clinics in Crohn's disease**

Xunchao Cai^1^, Nan Zhou^2^, Qian Zou^1^, Yao Peng^1^, Long Xu^1^, Lijuan Feng^1,*^, Xiaowei Liu^2,*^

^1^ Department of Gastroenterology and Hepatology, Shenzhen University General Hospital, 518055 Shenzhen, China

^2^ Department of Gastroenterology, Xiangya Hospital, Central South University, 410008 Changsha, China

**^*^Correspondence to:**

Lijuan Feng, Department of Gastroenterology and hepatology, Shenzhen University General Hospital, 518055 Shenzhen, China. Email: fenglj@szu.edu.cn; and Xiaowei Liu, Department of Gastroenterology, Xiangya Hospital, Central South University, Changsha, China. Email: liuxw@csu.edu.cn.

**Tables: 2**

**Figures: 6**

**Table captions**

**Table S1** Demographics characteristics of CD patients with mucosa samples collected

**Table S2** Characteristics of random forest model to separate CD patients and HCs using fecal bacteria

**Figure legends**

**Figure S1.** Alpha rarefaction curve based on Shannon diversity (a) and observed feature (b).

**Figure S2.** Pairwise PERMANOVA between groups based on unweighted unifrac distance. *, *p* ≤ 0.05; **, *p* ≤ 0.001; NS, differences not significant.

**Figure S3.** Error rate of random forest tree to separate different microbiota groups. (a). Error rate of random forest model between Uinf_M and Inf_M groups. (b). Error rate of random forest model between CD_F and HC_F groups.

**Figure S4.** Hierarchical clustering to detect sample outliers. Cut height was 40, and samples CD9, CD27, CD4 and CD5 were detected as outliers, which was removed in further analysis.

**Figure S5.** The correlation between co-occurrence taxa modules and the clinical traits. Numbers in the cells of left represent taxa numbers contained in the corresponding modules. The right color bar represents the module-trait correlation coefficient ranging from -1 to 1. The color in the cells of the middle columns represents the correlation marked by the right color bar, and numbers in the brackets were the *p*-value, numbers outside the brackets were correlation coefficient. Significant correlations were defined as *p* ≤ 0.05 and correlation coefficient ≥ 0.5 or ≤ -0.5.

**Figure S6.** Differences of the taxa abundance between the inflamed mucosa and uninflamed mucosa. Differences between the inflamed mucosa (Inf_M) and uninflamed mucosa (Uinf_M) based on the relative taxa abundance at the genus level (Two-sided Welch’s test, *p*-value ≤ 0.05 was considered significantly different, and only taxa showing differences higher than 0.1% were plotted on the figure).

A total of 54 CD patients and 14 HCs were included in this study. Totally, 38 fecal samples and 45 mucosal samples were collected from the CD patients. Demographic and clinical characteristics of the 52 participants with fecal samples collected have been reported in our previous work, of which strong positive correlation of SAS and SDS and weak positive correlation between anxiety disorders patients and disease severity were observed (Table S1).

| **Characteristics** | **Inf_M** | | **Uinf_M** | | **CD_F** | **HC_F** |
| --- | --- | --- | --- | --- | --- | --- |
| Sample counts, N | 22 | | 23 | | 38 | 14 |
| **Demographics** | |  |  |  |  |  |
| Age, median (mean±SD) | 28.77±9.48 | | 32.70±11.09 | | 30.16±10.89 | ﻿23.79 ± 2.08 |
| Gender, Male, n(%) | 15 (68.18) | | | 18 (78.26%) | 29 (76.32) | 6 (42.86) |
| BMI, kg/m^2^, (mean±SD) | 19.33±2.74 | | | 19.32±2.19 | 19.45±3.57 | NA |
| SAS | 38.70±7.28 | | | 40.83±7.66 | 38.82±9.70 | ﻿35.14 ± 4.09 |
| SDS | 41.66±10.34 | | | 43.90±10.33 | 41.66±11.83 | ﻿37.64 ± 6.28 |

Table S1 Demographics characteristics of CD patients with mucosa samples collected

NA represents not available.

Table S2 Characteristics of random forest model to separate CD patients and HCs using fecal bacteria

| OOB estimate of error rate: 11.54% | | | | | | |
| --- | --- | --- | --- | --- | --- | --- |
| Confusion matrix: | | | | Test data prediction | | |
|  | CD_F | HC_F | Class.error |  | CD_F | HC_F |
| CD_F | 35 | 3 | 0.07894737 | CD_F | 13 | 0 |
| HC_F | 3 | 11 | 0.21428571 | HC_F | 0 | 4 |


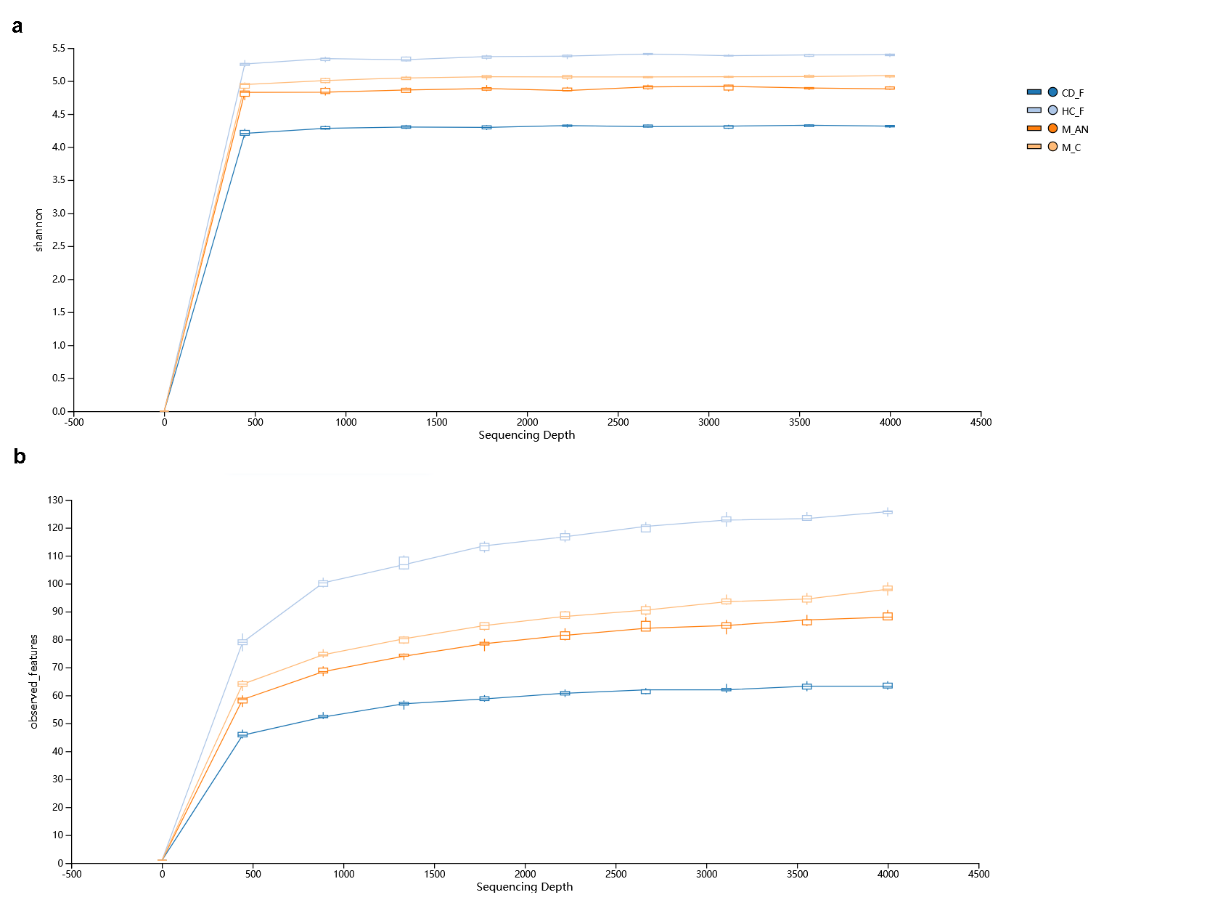


Figure S1. Alpha rarefaction curve based on Shannon diversity (a) and Observed feature (b).


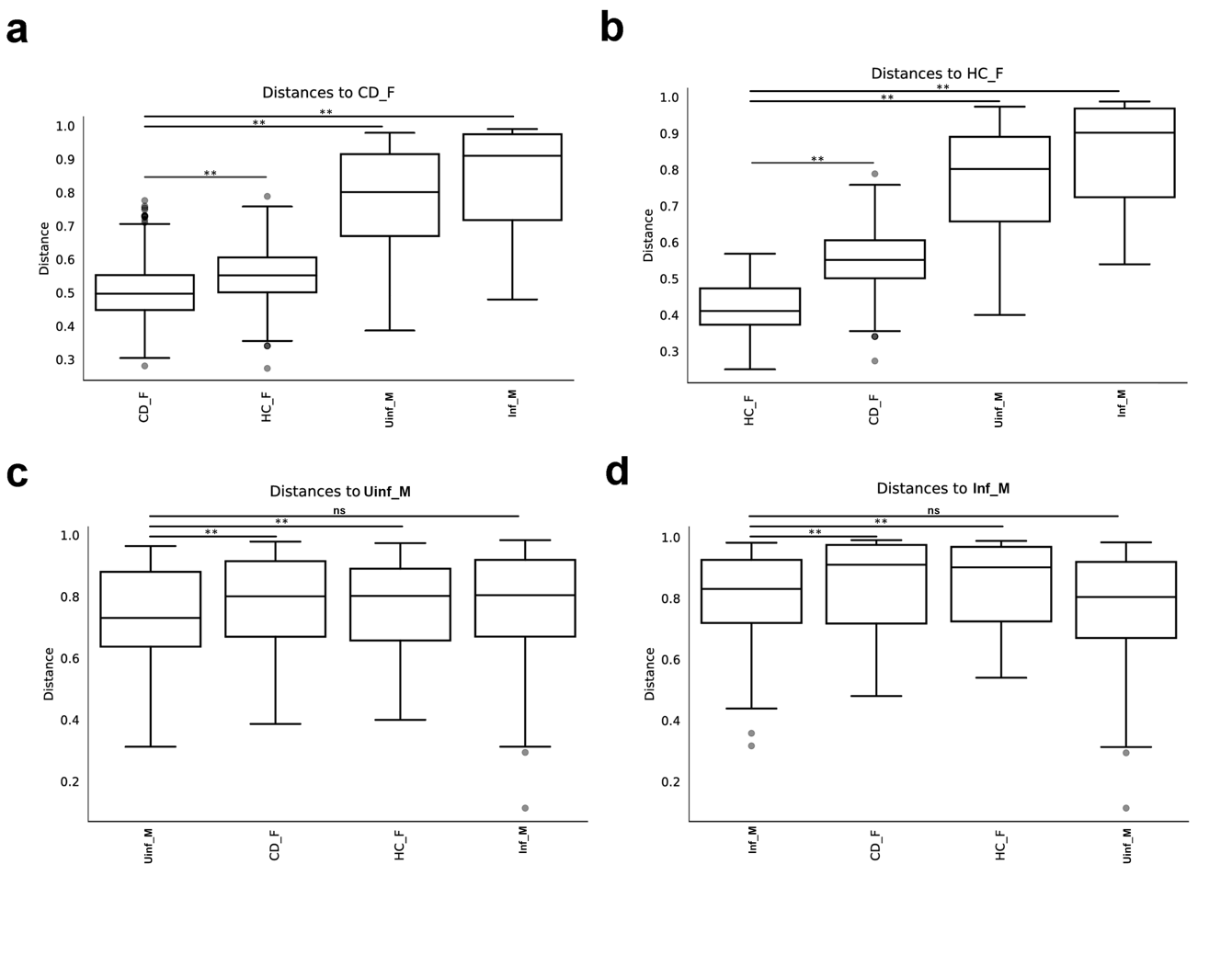


Figure S2. Pairwise PERMANOVA between groups based on unweighted unifrac distance. *, *p* ≤ 0.05; **, *p* ≤ 0.001; NS, differences not significant.


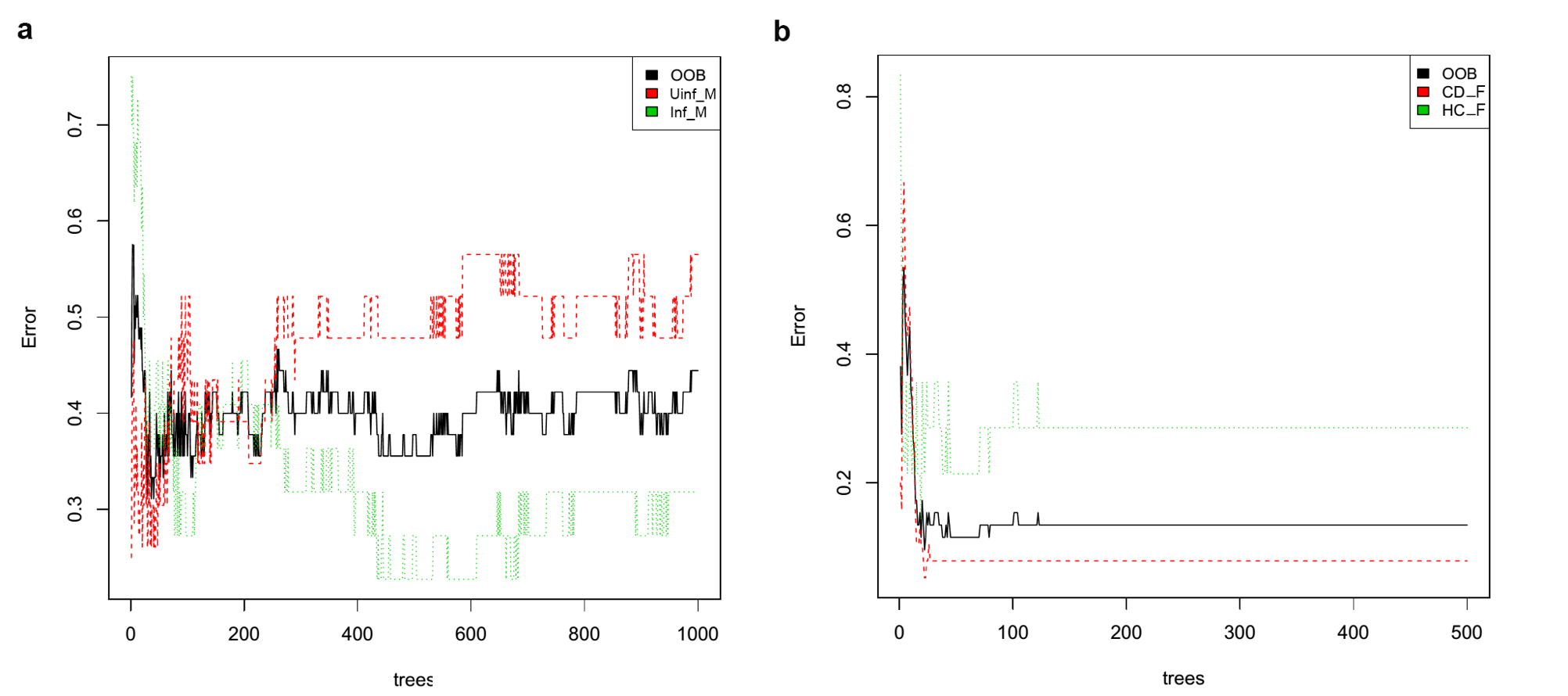


Figure S3. Error rate of random forest tree to separate different microbiota groups. (a). Error rate of random forest model between Uinf_M and Inf_M groups. (b). Error rate of random forest model between CD_F and HC_F groups.


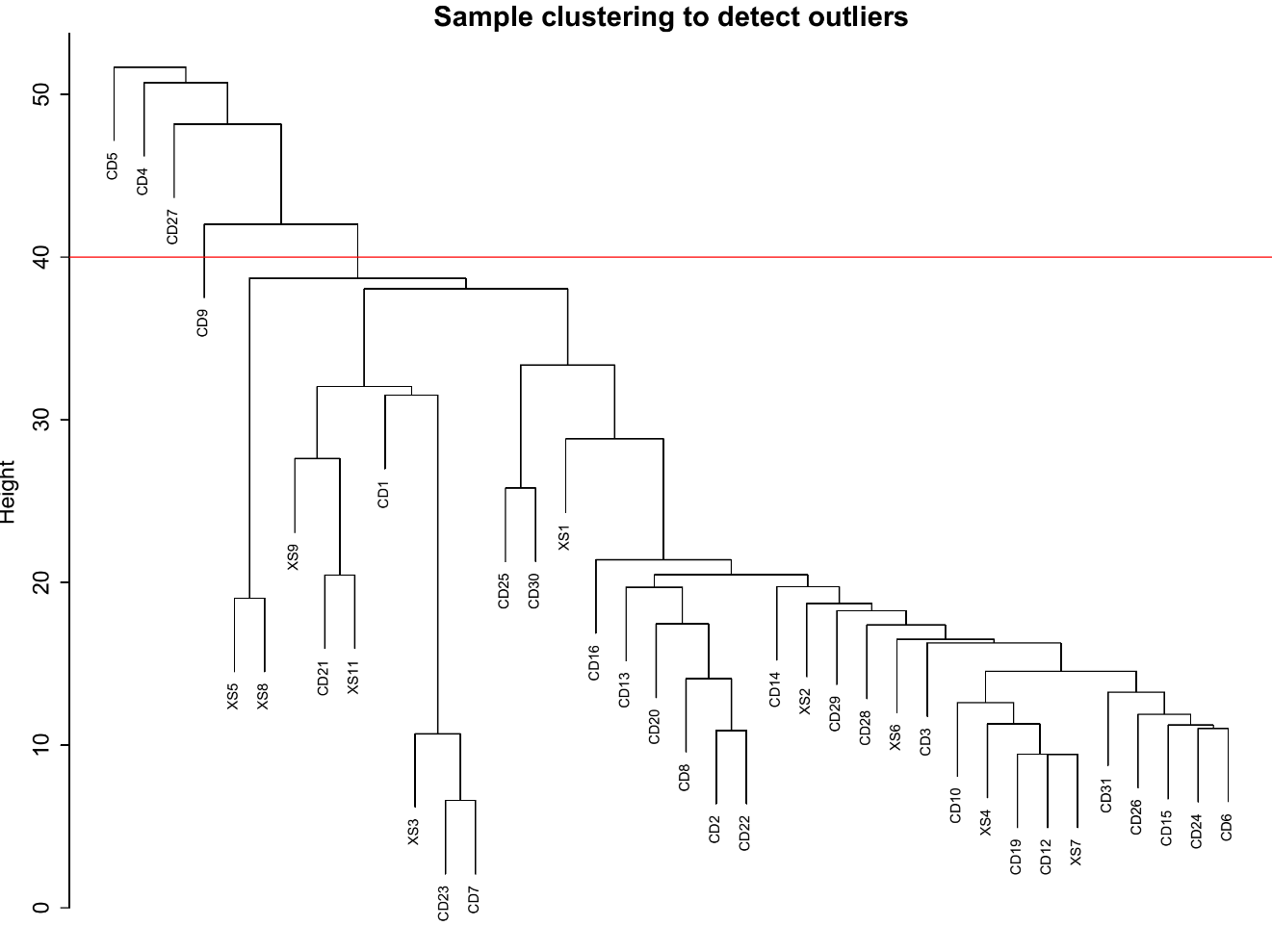


Figure S4. Hierarchical clustering to detect sample outliers. Cut height was 40, and samples CD9, CD27, CD4 and CD5 were detected as outliers, which was removed in further analysis.


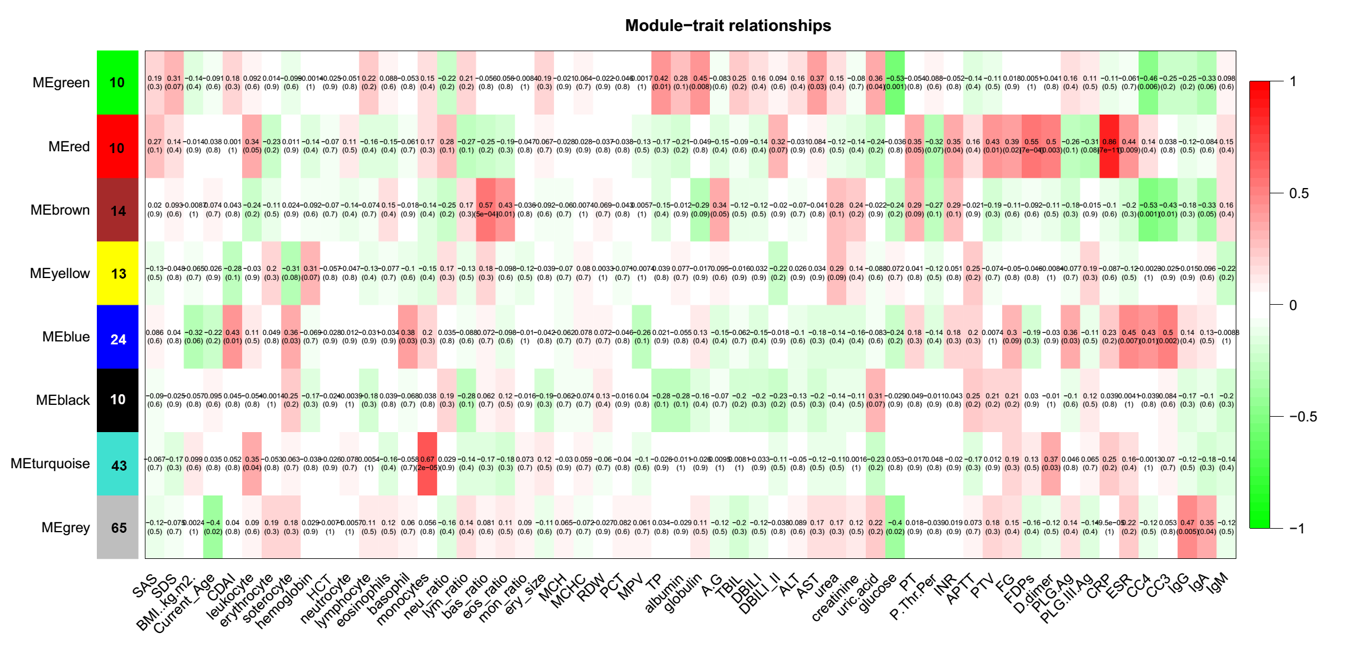


Figure S5. The correlation between co-occurrence taxa modules and the clinical traits. Numbers in the cells of left represent taxa numbers contained in the corresponding modules. The right color bar represents the module-trait correlation coefficient ranging from -1 to 1. The color in the cells of the middle columns represents the correlation marked by the right color bar, and numbers in the brackets were the *p*-value, numbers outside the brackets were correlation coefficient. Significant correlations were defined as *p* ≤ 0.05 and correlation coefficient ≥ 0.5 or ≤ -0.5.


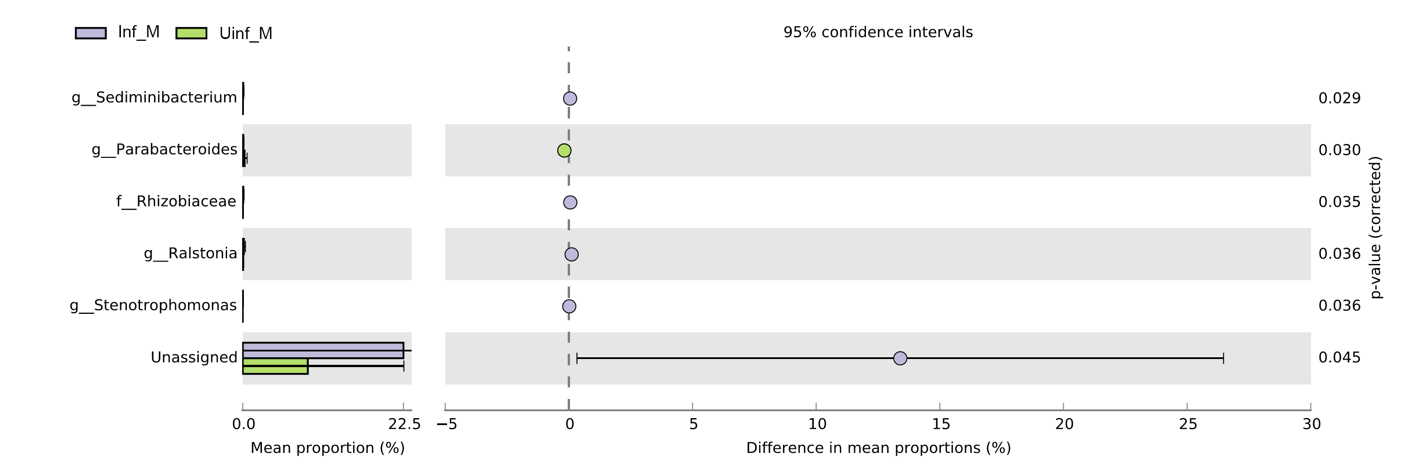


Figure S6. Differences of the taxa abundance between the inflamed mucosa and uninflamed mucosa. Differences between the inflamed mucosa (Inf_M) and uninflamed mucosa (Uinf_M) based on the relative taxa abundance at the genus level (Two-sided Welch’s test, *p*-value ≤ 0.05 was considered significantly different, and only taxa showing differences higher than 0.1% were plotted on the figure).
